# “Real-life” management of patients with severe asthma in the biologics era: Can we do better?^☆^

**DOI:** 10.1016/j.waojou.2021.100528

**Published:** 2021-03-18

**Authors:** Jeremy Charriot, Milka Maravic, Michael Huguet, Isabelle Vachier, Carey Suehs, Arnaud Bourdin

**Affiliations:** a Department of Respiratory Diseases, Univ Montpellier, CHU Montpellier, Montpellier, France; b University of Montpellier, PhyMedExp, INSERM, CNRS UMR, CHRU Montpellier, Montpellier France; c Rheumatology, Hôpital Lariboisière, APHP, Paris, France; d Real World Solutions, IQVIA, La Défense, France

**Keywords:** Asthma, Phenotype, Biomarkers, Biologic therapy

## Abstract

**Background:**

Discrepancies exist between guidelines and real-life practice in severe asthma. Objectives: To establish profiles for severe asthma patients according to their maintenance therapies and identify unmet needs.

**Methods:**

2432 French lung specialists and allergists were invited to participate in a severe asthma survey between March and April 2018. Retrospective data were collected using an electronic case report form developed by IQVIA.

**Results:**

71 respiratory physicians and/or allergists participated in the study, providing data for 736 severe asthma patients. The annual mean rates of hospitalization and exacerbation in the previous year were 0.65 (SD = 0.5) and 2.25 (SD = 1.0), respectively. One hundred one (13.7%) patients were treated with oral steroids; the mean dosage regimen was 16.1 mg per day (SD = 11.2). ICS-LABA-LAMA triple inhaled therapy was reported for 288 patients (39%); 231 patients (31.4%) had one biologic in their maintenance treatment. Among patients hospitalized at least once in the previous year (n = 311), 89 (28.5%) were currently treated with biologics, and 61 (19.6%) with oral steroids. One hundred sixty-six patients with uncontrolled asthma and no current biologic therapy had data for “T2 status”; 78 (47%), 89 (53.6%) and 137 (82.5%) of them had treatment criteria respectively for an *anti*-IgE, *anti*-IL5-pathway or *anti*-IL-4/IL-13 pathway therapy; 22 (13.2%) were ineligible for any current biologic according to biomarkers.

**Conclusion:**

Our study updated “real-life” therapeutic management data for severe asthma in France in 2018. We highlighted a need for improved patient-phenotyping. This work also gives a striking insight of the position of current and forthcoming biologics.

## Introduction

Asthma is a heterogenous disease whose main feature is chronic airway inflammation. Novel biologics such as IL5 and IL-4/IL-13 pathway-targeting therapies have made essential the phenotyping of severe asthma, according to GINA guidelines. This strategy aims to drastically decrease annual exacerbation rates and the need for oral steroids, while improving levels of asthma control.^1^, ^2^, ^3^These guidelines were mostly evidence based and potentially modulated by the recent ATS/ERS (American Thoracic Society/European Respiratory Society) task force where data robustness was confronted with grading strategies.^4^ Though these documents are considered as standard expert references by both patients and clinicians, in certain situations they can be challenged by payers. For example, whereas most studies have demonstrated significant benefits in patients with 2 exacerbations or more in the past 12 months, NICE (National Institute for Health and Care Excellence) in the United Kingdom complicated the decision-making process when they argued that biological therapies should be restricted to patients with 4 exacerbations or more per year.^5^

Beyond the various reactions to the evidence base for asthma care, the implementation of current recommendations by respiratory physicians is not always optimal.^6^^,^^7^ For instance, Cloutier et al showed that agreement with and adherence to asthma guidelines was low among both specialists and primary care clinicians for several key recommendations. Repeated epidemiological studies have also revealed that the overall level of asthma control in the general population is dramatically stable but poor.^8^^,^^9^ This observation was reinforced by pharmaco-epidemiological studies where oral corticosteroids (OCS) and short-acting beta-agonist (SABA) prescription refilling were used as surrogate markers of poor asthma control. It was shown that a large majority of severe asthmatics are buying a massive mean of 3.6 boxes of OCS every year (as a reminder, one box contains 20 pills of 20 mg each of prednisone equivalents) and were exposed to higher risks of death.^4^^,^^10^, ^11^, ^12^ Regarding SABA, the situation was shown to be no better: in the ambitiously large SABINA Swedish survey, SABA use was at extremely high rates and desperately associated with higher levels of mortality.^13^ Curiously, most currently available severe asthma cohort studies are driven by tertiary/expert asthma centers. In contrast, the above drug-use data suggests that a majority of these patients are managed outside these facilities.

Subsequently, real-world studies documenting asthma treatment strategies in situations unaffected by “NICE-like” prescription restrictions, and where the medication costs are not an issue for either the prescriber or the patient, take on increasing interest. This is the case for France, where several biologics have been sequentially introduced to the market between 2009 and 2019, and data concerning their usage should provide an insightful vision of the current level of asthma control, medication use and biomarker-elicited profiles. This likely helps at drafting tomorrow's landscape which can be extremely helpful for all the severe asthma stakeholders.

We then aimed to depict the profile and therapeutic management of severe asthma patients in the real world, identify unmet needs, and relate this to the use of current and futures therapies.

## Materials and methods

Two thousand four hundred thirty-two French lung specialists and allergists were invited to participate in a retrospective survey focusing on severe asthma between March and April 2018. Physicians were selected using OneKey, a regularly updated directory of 15 million healthcare professionals (HCP), 1 million healthcare organizations (HCOs) and their affiliations from over 100 countries.^14^ A minimum of 10 patients with severe asthma were selected per physician (the last 10 consecutive patients seen). Data were collected using an electronic case report form developed by IQVIA and validated by experts. The collection and processing of the information were based upon HCPs’ duly informed consent concerning the purpose of the study and their rights in accordance with general data protection regulation (GPDR) requirements. The data related to HCPs and patients were anonymously collected and analyzed.

### Study variables

Patient characterization included sex, age, employment status, and body mass index. Disease characteristics included the duration of severe asthma in years, asthma phenotype, blood eosinophil count before current maintenance therapy, type and dosage regimen of maintenance therapy. Atopy was defined by at least 1 positive skin prick test and/or a specific IgE concentration higher than 0.35 kUI/l directed against the commonest aeroallergens of the region.

The specialty, type of activity (liberal vs public hospital), and location for the diagnosing physician were also collected.

### Cost evaluation

In order to assess the hospitalization costs for asthma exacerbation, we obtained the overall hospitalization data for asthma occurring in France in 2018, (ie, n = 66,171 records from the French national claims databases^15^ with the primary diagnosis indicated by one of the following ICD-10 codes: J45.0, J45.1, J45.8, J45.9, and J46).^16^ We obtained the corresponding case mix of disease related groups (DRG) and applied the corresponding 2018 public costs to each DRG in order to obtain the mean per-patient hospital cost for asthma exacerbation (ie, 1414 euros).

### Statistical analyses

Descriptive statistics were provided, and differences in patient characteristics and maintenance therapies (GINA) were assessed using ANOVA tests for quantitative variables and Chi-squared or Fisher tests for qualitative variables. A bilateral p value < 0.05 was considered statistically significant. All calculations were carried out using the R statistical programming environment.^17^

## Results

Seventy-one respiratory physicians and/or allergists, of whom 63% worked part or full-time in a hospital environment, answered the survey. Physicians that responded were more likely to be men (71.8% versus 61.3% of non-responders) and less likely to work in the Paris area than non-responders (28.1% versus 21.3%) (Table S1– supplemental data).

### Patient characteristics

Data were provided for 736 patients with severe asthma. Among them, 54% were women. The mean age was 48.9 (SD = 16.44) years. The mean BMI was 25.9 (SD = 5.23) kg/m^2^. Fourteen percent of patients were unemployed, 52% had part- or full-time professional activity, 25% were retired, and 6% were students.

At the time of diagnosis, the mean FEV1/FVC ratio was 0.60 (SD = 12.2). Mean blood eosinophilia was 396 cells/mm^3^ (n = 515 patients). Atopy was reported in 83% of patients with severe asthma.

The annual mean rates of hospitalization and exacerbation in the previous year were 0.65 (SD = 0.90) and 2.25 (SD = 1.85), respectively. A history of admission into an intensive care unit was reported for 21% of patients. The most recent mean ACT score was 16.6 (SD = 4.4).

The percentages of never, active and former smokers were 55%, 13%, and 29.5%, respectively; 2.5% were using electronic cigarettes. Regarding comorbidities, 27% had allergic rhinitis, 22% gastroesophageal reflux disease (GERD), 19% anxiety disorders, 10% cardiovascular diseases, and 7% diabetes (Table 1).

**Table 1:** Patient characteristics.

	Patients (n = 736)
Age (SD)	48.9 (16.43)
Sex, Women (%)	397 (54)
Professional activity (%)	
Unemployed	103 (14)
Partial or full-time activity	383 (52)
Retired	184 (25)
Student	44 (6)
BMI (kg/m^2^, SD)	25.9 (5.23)
Eosinophil count at the time of diagnosis (cells per μL) (SD)	396 (261)
Missing data (%)	221 (30)
Mean pre-bronchodilator FEV1/FVC (SD)	0.60 (12.2)
Mean ACT score at the last evaluation (SD)	16.6 (4.4)
Number of exacerbations per patient in the past 12 months (SD)	2.25 (1.85)
Number of exacerbations per patient in the past 12 months resulting in hospitalization (SD)	0.65 (0.90)
Patients with ≥1 hospitalization in ICU in the past 12 months (%)	155 (21)
Diagnosis of allergic rhinitis (%)	199 (27)
Atopy (at least one positive SPT or specific IgE>0.15kU/l) (%)	611 (83)
Gastroesophageal reflux disease (%)	162 (22)
Cardiovascular diseases (%)	74 (10)
Diabetes (%)	52 (7)
Smokers (%)	
Active	93 (13)
Former	217 (29.5)
Never	408 (55)
E-cigarette users	18 (2.5)

ACT: asthma control questionnaire, BMI: body mass index, FEV1: forced expiratory volume in 1 s, ICU: intensive care unit, SPT: Skin Prick Test

### Severe asthma medication usage

One hundred one (13.7%) patients were treated with a maintenance daily dose of oral steroids with a mean dosage regimen of 16.1 mg per day (SD = 11.2) of prednisone equivalents. ICS-LABA-LAMA triple inhaled therapy was reported for 288 patients (39%). Two hundred thirty-one patients (31.4%) had one biotherapy in their maintenance treatment. Of note, 29 (4%) had no inhaled corticosteroids (Table 2). Among patients hospitalized at least once in the previous year (n = 311), 89 (28.5%) were treated with biologics, 61 (19.6%) with oral steroids, 14 (4.5%) with both biologics and oral steroids, and 72 (23.1%) had a triple inhaled therapy.

**Table 2:** Reported therapies of patients with severe asthma in 2018.

ICS + LABA + LAMA (%)	288 (39)
Any ICS + LABA (%)	697 (95)
ICS alone (%)	8 (1.1)
No ICS (%)	30 (4)
Antileukotriene agent (%)	329 (44.7)
Oral steroids (%)	101 (13.7)
mean dose per day (mg, SD)	16.1 (11.2)
Biologic (%)	231 (31.4)
Omalizumab	180 (24.5)
Mepolizumab	49 (6.7)
Benralizumab	1 (0.1)
Dupilumab	1 (0.1)
Thermoplasty (%)	7 (1)
Clinical trial (%)	13 (1.8)
Others (%)	86 (11.7)

Date are mean (SD), n, or n (%) - ICS: inhaled corticosteroid, LABA: long acting beta agonist, LAMA: long acting muscarinic agonist

In patients with available eosinophilia data before initiation of current therapy (515/736), 315 (61.2%) had a blood eosinophil count ≥300 cells/mm^3^; among them, 152 (29.6%) patients were admitted at least once during the last 12 months. Among the latter 52 (34.2% of hospitalized patients) were receiving biologics in their current maintenance therapy. These results are quite similar for patients with no history of admission to the hospital in the past 12 months, except for the proportion of patients treated with oral steroids, regardless of serum eosinophil count, which was less important in this group (40 patients, 9.4%) (Table 3, Table 4).

**Table 3:** Reported therapies of patients with severe asthma in 2018 and ≥ 1 admission to the hospital in the past 12 months.

≥1 admission:	n = 311/736
ICS + LABA + LAMA (% of patients hospitalized)	72 (23.2)
Oral steroids (% of patients hospitalized)	61 (19.6)
Biologic (% of patients hospitalized))	89 (28.6)
**≥1 admission and serum eosinophil count** ≥ **300/mm**^**3**^**:**	**n** = **152/515**
ICS + LABA + LAMA (% of patients hospitalized)	63 (41.4)
Oral steroids (% of patients hospitalized)	26 (8.4)
Biologic (% of patients hospitalized)	52 (34.2)

Date are mean, n, or n (%) - ICS: inhaled corticosteroid, LABA: long acting beta agonist, LAMA: long acting muscarinic agonist

**Table 4:** Reported therapies of patients with severe asthma in 2018 and < 1 admission to the hospital in the past 12 months.

<1 admission:	n = 425/736
ICS + LABA + LAMA (% of patients hospitalized)	158 (37.2)
Oral steroids (% of patients hospitalized)	40 (9.4)
Biologic (% of patients hospitalized))	142 (33.4)
**<1 admission and serum eosinophil count** ≥ **300/mm**^**3**^**:**	**n** = **163/515**
ICS + LABA + LAMA (% of patients hospitalized)	69 (42.3)
Oral steroids (% of patients hospitalized)	18 (11.0)
Biologic (% of patients hospitalized)	66 (40.5)

Date are mean, n, or n (%) - ICS: inhaled corticosteroid, LABA: long acting beta agonist, LAMA: long acting muscarinic agonist

### Economic burden

Among the 736 patients of the study, 311 (42.3%) were admitted at least once in the previous year, representing a total of 475 hospitalizations for a mean annual cumulative cost of 671,650 €/year. Among the 515 with available T2 biomarkers (blood eosinophil count and serum total IgE), 100 who were eligible for biologics (*anti*-IgE and/or *anti*-IL5) were hospitalized at least once in the previous year. The latter corresponded to a total of 136 hospitalizations representing a cost of 192,304€ (28.6% of the total hospitalization-related costs) (Table S2 **– supplemental data).**

### Indications for biologics

**Fig. 1:**
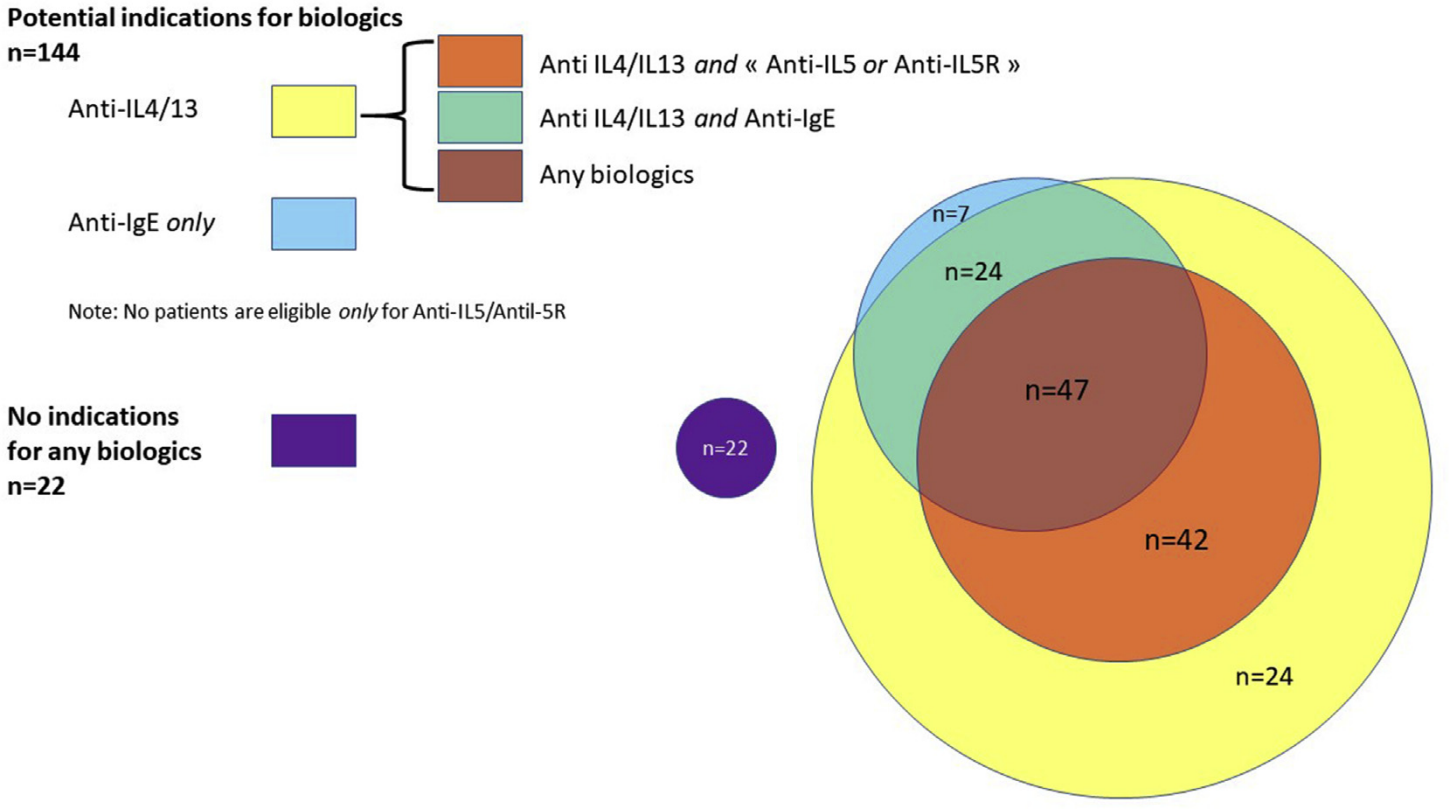
Proportion of patients with uncontrolled severe asthma but without any current biologic therapy (among patients with data for serum eosinophils and total IgE, n = 166)

Among the 424/736 patients with uncontrolled asthma (ACT<20 and ≥ 2 exacerbations in the past 12 months) despite optimal inhaled therapy, 305 had no biologics. Among these 305 patients, 166 had data indicating a “T2 status”; 78 (47%), 89 (53.6%), and 137 (82.5%) patients, respectively, had criteria for being treated an *anti*-IgE therapy, an *anti*-IL5 pathway therapy or an *anti*-IL4/-13 therapy. Interestingly, 22 patients had no indication for any biologic (Fig. 1).

## Discussion

This study describes severe asthma management in secondary or tertiary care in France in 2018. Demographic and clinical data are consistent with the recent literature.^1^^,^^3^^,^^18^ Exacerbation rates were lower in our study, most likely because only patients managed in secondary or tertiary care were enrolled.

Among the 736 patients, approximately three quarters had been “phenotyped” for T2 status and around one third were treated with biologics. The vast majority of the latter were treated with omalizumab, which is obviously due to its earlier development and commercialization.

According to the above observations, biomarker assessment is still not systematic in routine clinical practice (around 30% missing data for eosinophilia in our study). Eventually, the rise of FeNO evaluations, which are still not refunded in France and thus rarely performed, will increase awareness for the added-value of biomarkers in general. Historically, skin prick tests were the only biomarker of atopy used for characterizing asthma, and it seems that this part of history is still present.

One important issue highlighted by our study is the “under-management” in secondary care of the most severe patients who were recently hospitalized because of their asthma. Indeed, around 66% were eligible to receive an IL5-targeting drug but did not. More generally, a large proportion of them were not initiated with biologics despite being eligible and suffering from frequent exacerbations and poor asthma control. These observations are consistent with those made in primary care, in severe and non-severe patients. In the REALISE survey (8000 patients from 11 european countries), 45% of respondents had uncontrolled asthma.^19^ However, data are more sparse in regards with the management of severe asthma in secondary care, and much more concerning the use of biologics. An Italian Respiratory Society survey showed that despite uncontrolled asthma, treatment was stepped up by specialists in only 37.2% of the cases; asthma control questionnaires were only used in 65% of patients.^20^ In an attempt to explain the observed under-management in our study, we can mention OCS addiction, fears of biologics/shots, insufficient awareness, uncontrolled or poorly understood comorbidities, age or poor adherence including active smoking or non-removal of a clear triggering factor (cats are typical in this area). Of note, *anti*-IL-4/IL-13 therapy, which is not market-approved at this time in France, corresponds to the vast majority of indications for biologics in “under-treated” patients. This fact is certainly of interest when we think about how each therapy fits into an optimized decision-making process (ie, therapies that apply to wider group of patients are likely to be used more often).

Consequently, another insightful result of our study is the cost of severe asthma. Indeed, among 736 patients, we computed a potentially avoidable cost of 671,650€ mainly driven by the number of hospitalizations in patients with suboptimal therapeutic management according to current guidelines. Obviously, these results should be cautiously extrapolated to the general population of severe asthmatic patients as our conclusion relies on declarative data. In a recent case-control study using a national French medical claims database, the estimated medical direct cost of severe asthma was $9227/year versus $3950/year for matched patients without asthma.^21^ This does not include indirect costs for society such as sick leave, fear of professional/physical activity, or the management of corticosteroid adverse effects. From this perspective, we do think that no extreme restrictions should be made in regards with the prescription of biologics and that the inclusion criteria used in the studies should guide our therapeutic strategy. Aside from the obvious clinical benefit, this is a major argument for promoting a systematic and early assessment of these patients in expert centers with appropriate facilities and access to biomarkers and ongoing clinical trials and biologic therapies. This also might be an argument to discuss the current reimbursement price of biologics, as they will probably impose themselves as unavoidable.

There are several limits to this study that should be taken in account. First, as for any survey, there is an obvious potential for selection bias. Indeed, only 3% of the contacted physicians were recruited. This may be explained by the length and completeness of the questionnaire, which in turn provides us with precise and valuable information. In addition, the observed patient characteristics correspond well with those of the most recent randomized controlled double-blind trials.^6^^,^^8^ Secondly, approximately 30% of blood eosinophil counts were lacking (221 over 736), and so the number of patients eligible for biologics is likely underestimated. Lastly, our study snapshots severe asthma management in 2018, the year during which both mepolizumab and benralizumab were commercialized in France, and the landscape is likely to change with the forthcoming commercialization of dupilumab and tezepelumab.

## Conclusion

Our study updates data for “real-life” therapeutic management in a French severe-asthma population for the year 2018. We highlighted a need for better phenotyping and subsequent treatment tailoring, which could lead to a substantial improvement in public health costs, in addition to an obvious improvement in clinical outcomes. High rates of eligibility for biologics in approved indications suggests that the unmet need represented by low-T2 patients corresponds to only one third of the severe asthma population. Finally, there is clearly room for improving communication between severe asthma expert centers and secondary/primary care. The latter may be achieved through continuing medical education, active participation in multidisciplinary meetings or to the constitution of a register for severe asthma.

## Abbreviations

ACT, Asthma Control Questionnaire; FeNO, Fraction Expired of Nitric Oxide; FEV1, Forced Expiratory Volume in 1st Second; FVC, Forced Vital Capacity; GINA, Global Initiative for Asthma; ICS, Inhaled CorticoSteroids; IgE: Immunoglobulin E; IL5, Interleukin 5; IL5R, Interleukin 5 receptor; IL4/13, Interleukin 4/13; LABA, Long Acting Bêta Agonist; LAMA, Long Acting Muscarinic Agonist

## Availability of data and materials

The data that support the findings of this study are available from the corresponding author upon reasonable request.

## Ethics statement

No ethics approval was needed for this survey.

## Funding

No fundings supported this study.

## Declaration of competing interest

The authors have no conflicts of interest to declare in relation with this work. They all contributed equally to this work and give their consent for the publication of this article.
