# Supplementary material for: “Real-life” management of patients with severe asthma in the biologics era: Can we do better?
Source: World Allergy Organ J. 2021 Mar 18;14(4):100528. doi: 10.1016/j.waojou.2021.100528 (PMC7985558; doi:10.1016/j.waojou.2021.100528)
Supplement: Multimedia component 1 [file mmc1.docx]

**Supplemental data:**

**Table S1. Characteristics of survey responders**

| **Sex** | **Non responders** | **Responders** | **Total** |
| --- | --- | --- | --- |
| Women | 856 | 20 | 876 |
| Men | 1358 | 51 | 1409 |
| N/A | 147 |  | 147 |
| **Total** | **2361** | **71** | **2432** |
|  |  |  |  |
| **Medical Practice** |  |  | **Total** |
| Public | 1264 | 46 | 1310 |
| Mixed | 385 | 12 | 397 |
| N/A | 164 |  | 164 |
| Private | 548 | 13 | 561 |
| **Total** | **2361** | **71** | **2432** |
|  |  |  |  |
| **Region** |  |  | **Total** |
| Auvergne-Rhône-Alpes | 273 | 10 | 283 |
| Bourgogne Franche-Comté | 77 | 3 | 80 |
| Bretagne | 99 | 1 | 100 |
| Centre - Val de Loire | 65 | 1 | 66 |
| Corse | 11 |  | 11 |
| Grand Est | 203 | 6 | 209 |
| Hauts de France | 210 | 6 | 216 |
| Ile De France | 472 | 20 | 492 |
| Normandie | 119 | 5 | 124 |
| Nouvelle Aquitaine | 170 | 5 | 175 |
| Occitanie | 230 | 5 | 235 |
| Pays de la Loire | 90 | 2 | 92 |
| Provence Alpes Côte d'Azur (PACA) | 195 | 7 | 202 |
| N/A | 147 |  | 147 |
| **Total** | **2361** | **71** | **2432** |
|  |  |  |  |

*Data are expressed as n*

N/A: unknow data

**Table S2. Hospitalization-related costs over 1 year**

| **Patients with ≥1 hospitalizations in the previous year** | **Number of hospitalizations in the previous year in patients eligible for biologics** | **Hospitalization-related costs in patients eligible for biologics** |
| --- | --- | --- |
| 311/736 | 136/515 | 192 304€ |

Using the case mix of disease related groups (DRG) related to asthma and after applying the 2018 public costs per DRG, the mean hospital cost for asthma was 1,414 euros
